# A Missense Mutation c.1132G > A in Fumarate Hydratase (FH) Leads to Hereditary Leiomyomatosis and Renal Cell Cancer (HLRCC) Syndrome and Insights into Clinical Management in Uterine Leiomyomata

**DOI:** 10.3390/genes14030744

**Published:** 2023-03-18

**Authors:** Yue Shi, Yan Xu, Chao Wang, Yiqing Chen, Xiaojun Ren, Yu Kang, Chao Wang

**Affiliations:** 1 Obstetrics and Gynecology Hospital, Fudan University, Shanghai 200011, China; shiyue7467@fckyy.org.cn (Y.S.); xuyan7844@fckyy.org.cn (Y.X.); wangchao1519@fckyy.org.cn (C.W.); www.15301050258@fudan.edu.cn (Y.C.); xiaojunren@outlook.com (X.R.); 2 Shanghai Key Laboratory of Female Reproductive Endocrine Related Diseases, Shanghai 200011, China

**Keywords:** hereditary leiomyomatosis and renal cell cancer syndrome (HLRCC), fumarate hydratase (FH) gene, clinical management in hereditary gynecological disease, in vitro functional study

## Abstract

Background: HLRCC syndrome is a hereditary cancer predisposition syndrome caused by heterozygous germline pathogenic variant of the fumarate hydratase (FH) gene and characterized by cutaneous leiomyomas (CL), uterine leiomyomas (UL), and renal cell carcinoma (RCC). Loss of function variant of FH gene inactivates the Kreb’s cycle enzyme activity and predisposes individuals with such variant to the development of HLRCC. Methods: Next-generation sequencing (NGS) and Sanger confirmation were given to family members accessible. Following that, a functional study in vitro was performed to further confirm the pathogenicity of the variant. FH-Wild type (FH-WT) and FH-mutant (FH-MUT) (E378K) plasmid were constructed and transfected into 293T and uterine leiomyoma cell lines, respectively. Proliferation assessment was executed to show how this mutation affects the growth of uterine leiomyoma. qPCR and Western blotting were performed to investigate the change of transcription and translation of FH with mutation (E378K), and FH enzyme assay activity were tested in 293T cells with mutation and wild-type plasmids. Results: Here, we presented two families with the same missense variant (c.1132G > A) that has not been reported as a germline mutation in hereditary uterine leiomyomas before and classified as VUS in gene databases. Our in vitro experiments supported the pathogenicity of this missense variant, especially in uterine leiomyomata. Conclusions: According to the American College of Medical Genetics (ACMG) guideline, the E378K variant was classified as likely pathogenic (with evidence PS4_support, PS3_support, PM2_support, PP1, PP3 and PP4 evidence). Further insights into clinical management in uterine leiomyomata were discussed and should be practiced in gynecological clinical settings.

## 1. Introduction

HLRCC is an autosomal-dominant disorder that was first described by Launonen V et al. in 2001 [1]. It is characterized by cutaneous leiomyomas and uterine leiomyomas as the earlier features, while renal cell carcinoma (RCC) may develop at later stages. The absolute risk of developing RCC is estimated to be 10–15% [2,3]. Adrenal nodules and pheochromocytomas seem to be additional manifestations while further investigations would still be needed [3,4,5]. Cutaneous leiomyomas as one of the most common clinical features presented in over 80% of HLRCC patients, while studies implied that penetrance of cutaneous leiomyomas was low in Japanese, Korean, and Chinese HLRCC patients compared with Caucasians [6,7]. Females usually had uterine leiomyomas at the age of 30–50 years old, while females with HLRCC could develop multiple leiomyomas at a much younger age at 28–32 years old, with large and numerous myomas specifically [8,9]. As uterine leiomyoma is the most high-penetrance trait of HLRCC, as 80–90% of women with HLRCC would be diagnosed with uterine leiomyoma in their lifetime, which is characterized by early onset, multiple, large, and symptomatic myomas, ultimately resulting in myomectomy or finally hysterectomy. To be noticed, the pathological examination results are typically benign. Bennett et al. [10] collected the morphologic features of 31 cases of FH-deficient ULs and found that FH deficient ULs could be initially excluded based on specific morphological characteristics (“FH-d morphology”) shown under low and high magnification [9,10,11,12,13,14,15,16,17]. The features were concluded as follows: prominent branching blood vessels with thin walls (also: “staghorn” or “hemangiopericytoma-like” vessels) [13], a specific edematous pattern (“alveolar”) [10], rhythmic pattern of cellular arrangement (“chain-like”) and bizarre nuclei under low magnification [10,15], and eosinophilic cytoplasmic inclusions and huge nuclei (“macronucleoli”) with perinucleolar halos under high magnification [11]. Renal cell cancer attacks HLRCC patients at the median age of 40 to 41 years old [9], and the renal cell cancers in this syndrome were mostly papillary type II RCC (PRCC2), which was often solitary, unilateral, and extremely aggressive.

FH gene pathogenic variant could lead to HLRCC in monoalleic carriers and FHD in bialleic carriers [18]. FH gene consists of ten exons encompassing 22.15 kb of DNA and is highly conserved across species. FH is a tumor suppressor gene located at chromosome region 1q42.3-43 which encodes the enzyme fumarate hydratase, which plays a critical role in both the tricarboxylic acid (TCA) cycle in mitochondria and DNA double-strand breaks in the nucleus [1,19]. Because of the mutation in the FH gene, fumarate hydratase activity will be reduced or absent. This increases the levels of intracellular fumarate which inhibits the activity of hypoxia-inducible factor (HIF) prolyl hydroxylases, contributing to the accumulation of HIF-α. HIF-α further activates the HIF pathway, which is closely related to tumor growth and metastasis [20,21].

Fumarate hydratase deficiency (FHD) caused by biallelic alterations of the FH, by the hereditary mode of autosomal recessive, is a rare disorder of the tricarboxylic acid cycle, classically characterized by encephalopathy, profound psychomotor retardation, seizures, a spectrum of brain abnormalities and early death in childhood. Less common milder phenotypes with moderate cognitive impairment and long-term survival have been reported.

Here, we report a FH missense mutation c.1132G > A (p.E378K) in exon 8 that was never documented before as a germline mutation associated with HLRCC, that upgraded as likely pathogenic based on segregation analysis, molecular characterization of blood and tumors, and further in vitro functional study, which extended our knowledge about the pathogenic mutations of FH in an attempt to delineate the role of this gene in pathogenicity and carcinogenesis.

## 2. Case Presentation

### 2.1. Case 1

A 32-year-old female showed with menostaxis (period usually lasts for 11 to 12 days) for about half a year. Menarche occurred at 13 years of age, and her menstrual cycles had been regular till half a year ago. Pelvic ultrasound showed multiple hypoechoic masses with the maximum size of 104 mm × 87 mm × 64 mm, protruding into the uterine cavity. Previously, she was diagnosed with multiple uterine leiomyomas at 23 years old and underwent a laparoscopic myomectomy. However, an ultrasound revealed a recurrence 5 years after surgery with the maximum diameter of the leiomyoma at about 5 cm. She was diagnosed with renal cell cancer at the age of 30 and received a radical nephrectomy. The tumor was reported as papillary renal cell carcinoma type II (pT1aN0M0) pathologically. IHC-staining showed negative for FH. Considering her suspicious medical history and our doubt of the possibility of HLRCC (germline mutation of FH), a genetic test was executed, and she was found to have mutation c.1132G > A (p.E378K) in exon 8 of FH with uncertain significance. As to the patient’s demand, a hysterectomy was performed this time. The final pathologic report proved the uterine leiomyoma lesions to be FH-deficient as expected.

**Figure 1:**
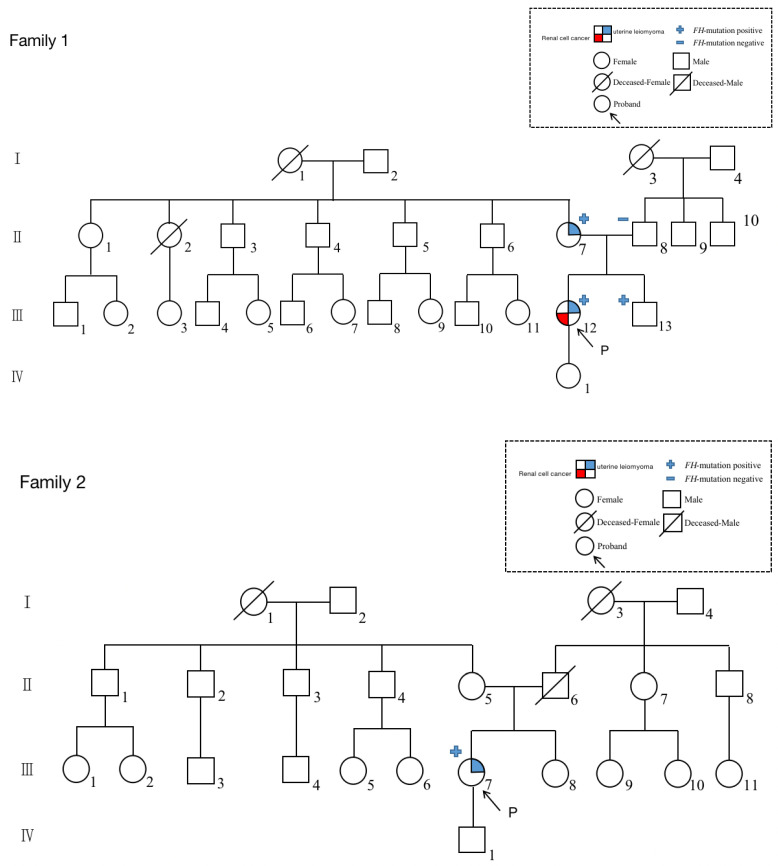
Family tree.

**Figure 2:**
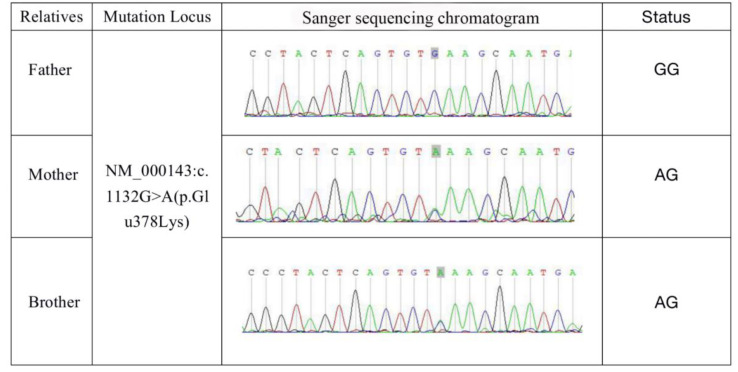
Sanger sequencing test results of Case 1. The spot mutation (gray square) was tested in family members accessible of Case 1.

A gene test was given for the family members accessible and family tree (a family of four generations) was drawn as Family 1 in Figure 1. The proband, our patient (III-12), was diagnosed with uterine leiomyoma at age 23, and renal cell cancer at age 30. Her mother (II-7) was diagnosed with uterine leiomyoma in her 20s. After genetic counseling, three family members agreed to perform a sanger test for the FH variant. Her mother (II-7) and her brother (III-13) were confirmed with the same FH mutation (Figure 2). Given the variant co-segregation and cancer history, PP1 evidence could be applied for upgrading this FH VUS variant.

### 2.2. Case 2

**Figure 3:**
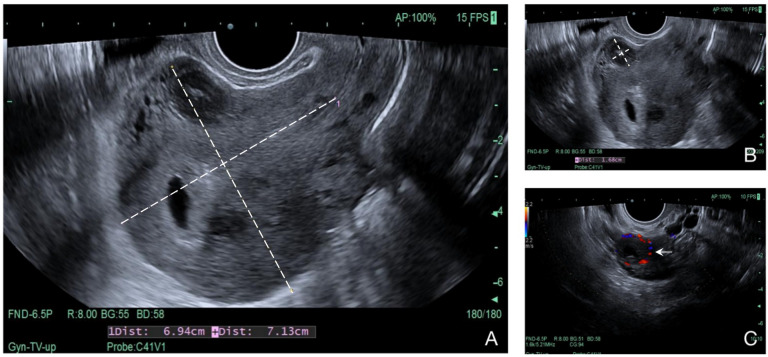
Ultrasound Results of case 2. (**A**) Uterine size showed by ultrasound. (6.94 cm × 7.13 cm). (**B**) The maximum size of leiomyomas (16 mm × 17 mm × 12 mm). (**C**) Extensive blood supply for the uterine leiomyomas.

**Figure 4:**
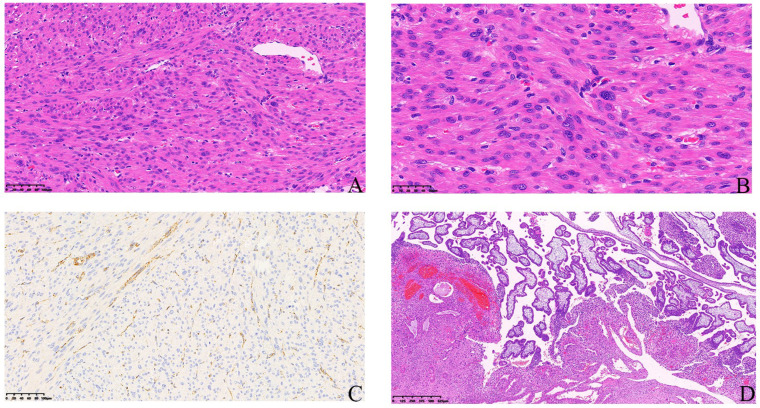
Results of immunohistochemistry of uterine leiomyoma with fumarate hydratase (FH) deficiency. (**A**,**B**) Hematoxylin-eosin staining of a uterine leiomyoma biopsy (20×) (40×). (**C**) Cells of uterine leiomyoma were negative to FH, while adjacent vascular endothelial cells and vascular smooth muscle cells were positive to FH. (**D**) Leiomyomas with pregnancy.

A 30-year-old Chinese female presented with multiple myomas treated by myomectomy, and the final pathologic reports proved the lesions to be leiomyomas with bizarre nuclei with FH status unclear (did not obtain testing in the local hospital). However, pelvic ultrasound reported the recurrence of multiple myomas 3 months later on a follow-up visit. Then, the patient was further treated with GnRH agonist (GnRH-a) therapy for three months and the size of the leiomyomas decreased sharply. However, leiomyomas grew back rapidly since off GnRH-a for 2 months as shown in the ultrasound (Figure 3). She decided to receive a hysterectomy this time, and IHC-staining showed negative for FH (Figure 4), so she was advised to obtain a genetic test to exclude germline mutation. The same as the patient in case 1 mentioned above, mutation c.1132G > A (p.E378K) in exon 8 of FH, was identified for her.

After further investigation of the family history (Family 2 in Figure 1), the patient’s grandmother was found to have received a hysterectomy at a young age for multiple uterine leiomyomas, and her grandmother’s sisters were diagnosed as fibroids as well. However, her family members refused genetic testing for personal reasons.

## 3. Materials and Methods

### 3.1. Case Selection

Patients with typical clinical phenotype of HLRCC were selected Although diagnostic criteria for HLRCC have not been established, Smit et al. [22] have listed practical criteria for the clinical diagnosis of HLRCC. Histopathologically-confirmed cutaneous leiomyomatosis was proposed as a major criterion, and minor criteria includes:

(1) Severely symptomatic uterine leiomyomas treated with surgery before the age of 40, especially those confirmed to be FH-absent.
(2) Type 2 papillary or collecting duct renal cell carcinoma before the age of 40.
(3) Positive family history: A first-degree family member who meets one of the criteria. (Occurrence of severely symptomatic uterine leiomyomas < 40 years old in second-degree paternal family members may also be relevant.)

HLRCC can be suspected when an individual meets >= 2 of above-mentioned minor criteria. Two patients selected and displayed here were clinically diagnosed with HLRCC and patients were from Anhui and Zhejiang Province, which are in southeastern China for Case 1 and 2, respectively. The study was conducted according to the Declaration of Helsinki principles. Family members who participated in the study gave their written informed consent to disclose their clinical information and genetic information.

### 3.2. Detection of FH Mutations

Genetic tests were executed on patients and their family members as described above. QIAamp DNA blood kit (QIAGEN, Venlo, The Netherlands) was used to extract genomic DNA from peripheral blood. The obtained DNA was applied to a two-step polymerase chain reaction (PCR) as a template. For the first PCR amplification step, all coding exons from the 16 genes (MLH1, MSH2, MSH6, PMS2, POLE, EPCAM, STK11, PTEN, POLD1, TP53, BRCA2, BRCA1, MSH3, PMS1, FH and APC) were amplified simultaneously by multiplex PCR in one tube. The gene panel applied is for patients with uterine tumors developed by Shanghai Biotechnology Corporation. Secondary PCR amplification was performed using the first PCR product as a template. The purified PCR products were then applied to a NGS system (NovaSeq platform) using the NovaSeq Reagent Kit (Illumina, San Diego, CA, USA) according to the manufacturer’s instructions. The obtained data were further analyzed. The primer sequences for FH amplification were provided in the Supplementary Materials. The genetic test was approved by the medical ethics committee of the Obstetrics and Gynecology Hospital of Fudan University (Ob&Gyn Hospital) (Approval Number: 2020-45) and conducted in accordance with the Declaration of Helsinki.

### 3.3. In Silico Characterization of the Variant

The pathogenicity of FH missense mutation c.1132G > A (p.E378K) in exon 8 was analyzed from the effects on mRNA splicing and protein translation, respectively, through in silico analysis tools. The following tools were used [23,24,25,26]: for mRNA splicing, HSF (The Human Splicing Finder system), varSEAK website, a splice site prediction tool, that provides information about genetic variants from public databases, (https://varseak.bio/index.php, accessed on 17 March 2023) and SpliceAI, a deep-learning-based tool to identify splice variants were utilized; for the potential influence on protein, PolyPhen2 (Polymorphism Phenotyping version 2; http://genetics.bwh.harva rd.edu/pph2/, accessed on 17 March 2023) and Provean (Protein Variation Effect Analyzer; http://prove an.jcvi.org/index.php, accessed on 17 March 2023) were applied for the potential influence on protein. PolyPhen2 is a sequence and structure-based methodology that evaluates the structural and functional consequences of nsSNPs [27]. The range of scores estimated is from 0 to 1. The nsSNPs could be categorized as ‘possibly damaging’ and ‘probably damaging’ (>0.5) or ‘benign’ (<0.5). Provean evaluates the potential harm of variations on protein sequences [17]. A score equal to or below the threshold of −2.5 is defined as a deleterious non-synonymous single nucleotide polymorphism (nsSNP).

The SOPMA database was accessed to inspect the mutation effects on the secondary structure of protein. The SWISS-MODEL was used to build a three-dimensional structure model and visualization was realized with tool PyMOL.

### 3.4. In Vitro Functional Studies

#### 3.4.1. Construction of FH-Wild Type (FH-WT) and FH-Mutant (FH-MUT) (c.1132G > A) Plasmid

The plasmid was constructed with pLVX-EGFP-IRES-puro (Addgene plasmid # 128652) as a vector, for which it contained anti-Puromycin and green fluorescent protein (GFP). Mutations were created using the Quick-change kit (Agilent Technologies, Santa Clara, CA, USA) according to the manufacturer’s instructions.

#### 3.4.2. Cell Cultures and Transfection

**Uterine leiomyoma cell line and 293T cell line**.

Uterine leiomyoma cell lines were obtained from myomectomy (pathology reported regular uterine leiomyoma) and cultured in medium with DMEM (Dulbecco’s Modified Eagle Medium), 20% FBS and penicillin/streptomycin. The 293T cells were cultured in DMEM with 10% fetal calf serum.

Plasmid transfection.

Modified FH plasmid and package plasmid (psPAX2; pMD2.G) were transfected together with VigoFect to uterine leiomyoma cell lines. The virus concentrate was added to the uterine leiomyoma cell line and puromycin was added three days later to screen for the cells successfully transfected.

FH-WT and FH-MUT (c.1132G > A) plasmids were transiently transfected into 293T cell lines, respectively. Transfection was carried out using Lipofectamine 2000 reagent (InvitrogenLife Technologies) according to the manufacturer’s instructions.

#### 3.4.3. Proliferation Assessment

The cells post-selection of puromycin were seeded into a 96-well plate, and CCK8 was used to assess the cell proliferation in the 3 cell lines prepared, blank uterine leiomyoma cell line as control, the cell line with FH-WT plasmid transfected and the cell line with FH-MUT plasmid transfected.

#### 3.4.4. RNA Extraction, cDNA Synthesis, and Real-Time Quantitative Polymerase Chain Reaction (qPCR) Analysis

RNA extracted from 293T cells 48 h post-transfection with FH-MUT plasmid and FH-WT plasmid as control was used for quantitative reverse transcription PCR (RT-qPCR). RNA extraction was executed using TRIzol^®®^ (ThermoFisher Scientific, Waltham, MA USA) and RNA quality was evaluated by agarose gel electrophoresis; all samples had a 260:230 ratio above 1.90, measured using a NanoDrop (ThermoFisher Scientific). Total RNA (1 µg/µL) was reverse-transcribed with oligo(dT) primers using SuperScript™ III First-strand Synthesis System (ThermoFisher Scientific). FH primers for RT-qPCR were as following: Forward 5′-ATCCACGCTGTTTTGACCTC-3′; Reverse 5′- AGGAATTTTGGCTTGCCATT-3′.

#### 3.4.5. Western Blot Analysis

293T cells transfected with FH-MUT plasmid and FH-WT plasmid as control were lysed in RIPA buffer (Pierce) and protein concentration was measured by BCA Protein Assay Kit (Pierce). Antibodies (Santa Cruz Biotechnology, 1:5000) were used to target FH protein.

#### 3.4.6. Measurement of FH Enzyme Activity

Protein quantification in the extract was undertaken by the bicinchoninic acid colorimetric assay according to the Fumarase Activity Colorimetric Assay Kit (Biovision, Milpitas, CA, USA).

#### 3.4.7. Statistical Analysis

The statistical analysis was executed using IBM SPSS Statistics version 20. Continuous variables with normal distribution were presented as mean (standard deviation [SD]) for analysis. The differences of mean FH mRNA level between cells transfected with FH-MUT (c.1132G > A) and FH-WT as controls were compared using the Mann–Whitney U test. The same statistical analysis was executed to compare the enzyme activity between these two groups. A value of *p* < 0.05 was considered significant.

## 4. Results

### 4.1. Identification of FH Mutation in Patients

As described in the case presentation, both patients as proband in the family were found to be with missense mutation c.1132G > A of FH with no other mutation detected. To investigate the inheritance of FH mutation in the family, further genetic tests were conducted for their family members who were accessible (Figure 2). To clarify the causative role of the FH mutation c.1132G > A in HLRCC, in vitro experiments were further performed using mutant c.1132G > A-expressing cells and compared with the results of FH-WT cells.

### 4.2. The Missense Mutation c.1132G > A of FH Is Predicted to Be Highly Damaging

**Figure 5:**
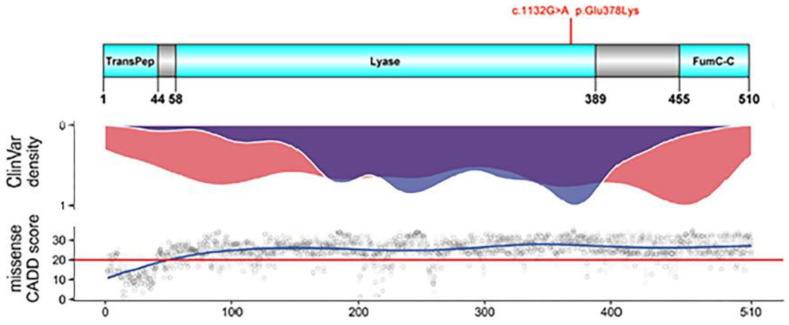
Mutation variant properties. Upper panel: Schematic representation of the FH protein, domains (ticks below *x*-axis numbered after NP_000134.2 and P07954), and localization of mutation E378K. TransPep, mitochondrion transit peptide; Lyase, N-terminal fumarate lyase domain.

The functional domain of FH consists of TransPep, mitochondrion transit peptide; Lyase, N-terminal fumarate lyase domain; FumC-C, C-terminal fumarase C domain as shown in the upper panel of Figure 5. E378K is apparently located in the region of lyase.

Lower panel: Density plot showing the distribution of (likely) pathogenic truncating (red) and missense (blue) variants reported in the publicly available database ClinVar which collects user submitted curations for variant pathogenicity.

For mRNA splicing, HSF (The Human Splicing Finder system), varSEAK website, a splice site prediction tool that provides information about genetic variants from public databases, (https://varseak.bio/index.php, accessed on 17 March 2023) and SpliceAI, a deep-learning-based tool to identify splice variants were utilized; for the potential influence of protein, PolyPhen2 (Polymorphism Phenotyping version 2; http://genetics.bwh.harvard.edu/pph2/, accessed on 17 March 2023) and Provean (Protein Variation Effect Analyzer; http://prove an.jcvi.org/index.php, accessed on 17 March 2023) were applied.

**Figure 6:**
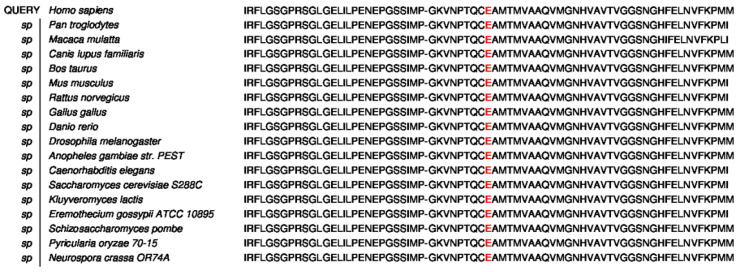
The missense mutation c.1132G > A (p.E378K) in exon 8 is highly conserved among 17 species.

The missense mutation c.1132G > A in exon 8 is highly conserved among 17 species, as shown in Figure 6. To predict the disease-causing effect of this mutation, in silico analysis with two prediction tools for protein function (PolyPhen2 and Provean) were performed as shown in Table 1. Both these prediction tools predicted the E378K mutation as a highly damaging amino acid substitution. Three prediction tools for mRNA splicing (HSF, varSEAK website and SpliceAI) used predicted that the mutation would likely change the donor spot for mRNA splicing. Although the mutation was predicted to be likely pathogenic by diverting the donor spot in HSF and SpliceAI, the varSEAK website predicted that it would be a tolerated mutation.

Another theoretical approach useful in the classification of FH germline missense variants is structural modeling.

**Figure 7:**
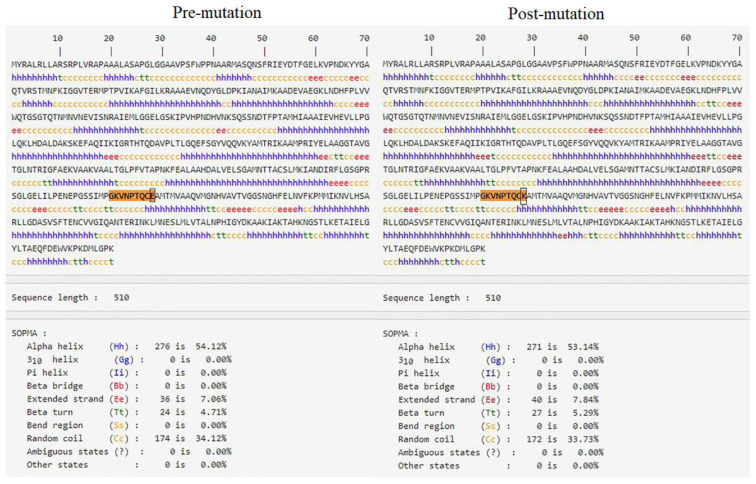
The predicted effects on the secondary structure by SOPMA database.

As the mutation took place in the fumarate lyase region, the potential effects on secondary structure were further inspected. According to SOPMA database, the structure regions, 310 helix (Gg), Pi helix (Ii), β bridge (Bb), Bend region (Ss) and Ambiguous states were spared and the structure changes in α helix (Hh), Extended strand (Ee), β turn (Tt), and Random coil (Cc) due to E378K mutation were displayed in Figure 7 in details. The structural changes of secondary structure caused by E378K mutation of Fumarate hydratase were influential.

With the tool SWISS-MODEL and PyMOL, we made the prediction of the effect of a given mutation on the protein conformation. Structural models with mutations are usually built on the scaffold of the crystal structure of the corresponding wild-type protein; as an alternative, the three-dimensional structure of a homologous protein should be used as template.

**Figure 8:**
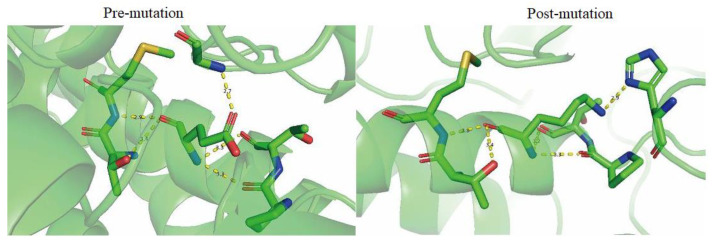
The predicted effects on the three-dimensional structure visualized by PyMOL.

E378K mutation was the mutation for which a disruptive effect on the protein conformation was predicted (Table 2). As shown in Figure 8, the distance of hydrogen bond in between M382 and E378, and T375 and E378 was profoundly altered post-mutation, which indicated a diversion of the structure of Fumarase lyase main chain. Additionally, the hydrogen bond between G144 and K378 broke and formed a new hydrogen bond in between H235 and K378 with a significant change in the distance, indicating the side chain structure also transformed post-mutation (Table 2, Figure 8). This dramatic structural conversion likely caused a loss of the protein active conformation.

### 4.3. In Vitro Functional Studies

#### 4.3.1. The c.1132G > A Mutation Increases the Cell Proliferation in Uterine Leiomyoma Cell

The cell proliferation in the uterine leiomyoma cell line, the cell line with FH-WT plasmid, and the FH-MUT plasmid transfected were evaluated (Figure S1) and compared. The cell proliferation in FH-MUT was found to be significantly higher than FH-WT and the control cell line, while no remarkable difference was shown between FH-WT and the control cell line (Figure S2).

#### 4.3.2. The E378K Mutation Does Not Influence the mRNA Transcription of FH

**Figure 9:**
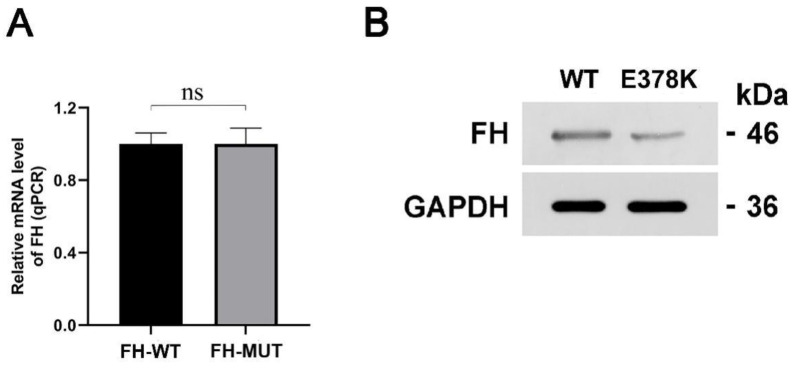
mRNA transcription (**A**) and FH enzyme expression (**B**) affected by E378K mutation.

Regarding mRNA expression of the housekeeping gene (GAPDH), there was no significant difference between cells transfected with FH-MUT and FH-WT plasmid (0.9996 ± 0.0611 vs. 1.000 ± 0.0088, *p* = 0.31, Mann-Whitney U test) as shown in Figure 9A.

#### 4.3.3. The Mutation Decreases the FH Enzyme Expression

We then asked whether the protein expression of FH would be affected by the FH missense mutation. As the mutation was a single nucleotide variant (SNV) (c.1132G > A), FH protein molecular weight predicted was 46 kDa. The FH protein was harvested from the cell cultures transfected with FH-MUT and FH-WT plasmid. The enzyme was significantly down-regulated with FH-MUT transfected (Figure 9B).

#### 4.3.4. The E378K Mutation Is Associated with Altered FH Enzyme Activity

**Figure 10:**
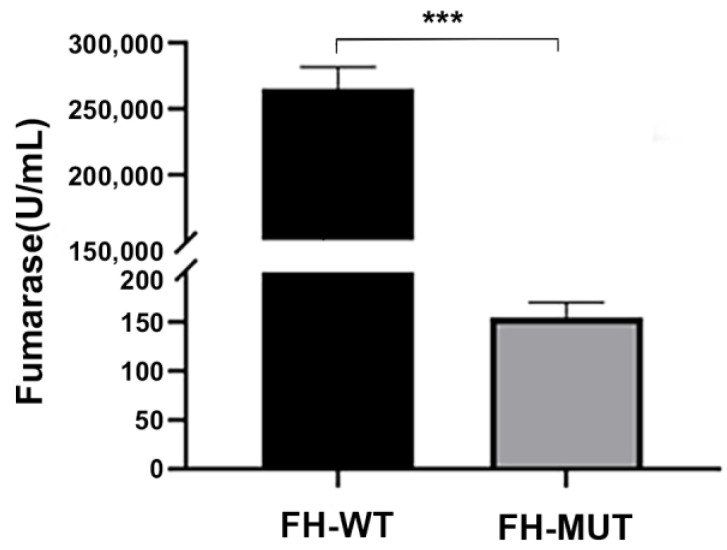
FH enzyme activity altered by E378K mutation. *** *p* < 0.001.

Whole-cell FH enzyme activity of 293T cell lines transfected with FH-MUT plasmid was significantly lower than whole-cell FH enzyme activity of cell lines with FH-WT plasmid (*p* < 0.001, Wilcoxon rank-sum test) (Figure 10). The mean FH enzyme activity of FH-WT cell-line was 2.6520 × 10^6^ U/mL, which was significantly lower compared with FH-MUT cell-line as 154.0827 U/mL. The enzyme activity in cells with missense mutation (c.1132G > A) was shown drastically decreased compared with the FH-WT cell line.

This section may be divided by subheadings. It should provide a concise and precise description of the experimental results and their interpretation, as well as the experimental conclusions that can be drawn.

## 5. Discussion

Inheritance of a single variant FH allele predisposes the individual to develop manifestations of HLRCC, while inherited biallelic pathogenic variants cause fumarate hydratase deficiency (FHD) in autosomal recessive mode, a disorder characterized by neurological impairment and death in the first decade of life [28].

**Figure 11:**
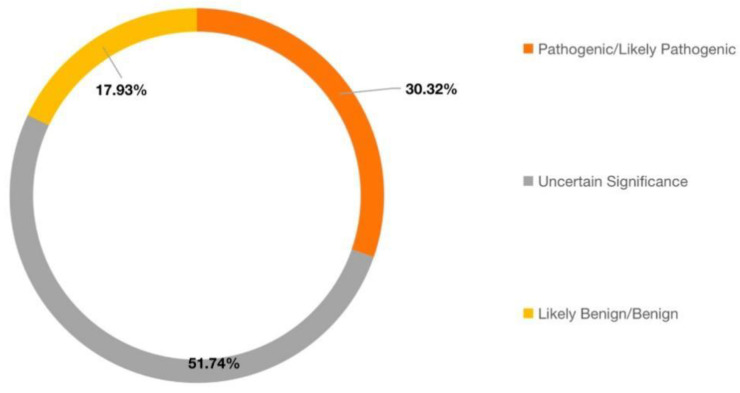
The distribution of pathogenicity for FH mutations in ClinVar database.

Currently, approximately 30% of FH variants are considered to be pathogenic or likely pathogenic in ClinVar (May 2021). About 52% of variants were classified as ‘Uncertain Significance’ and further studies were urgently in demand (Figure 11). The missense variant accounts for the largest proportion in VUS according to the FH mutation database [27].

**Figure 12:**
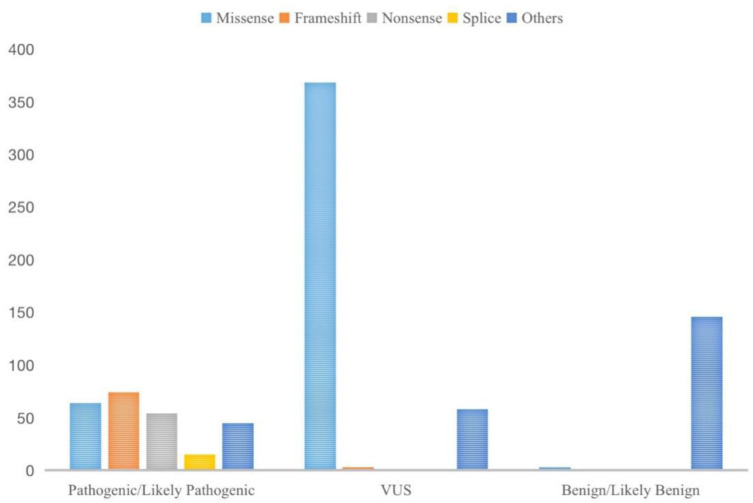
The proportion of different variants in FH sorted by clinical significance and different variant types in pathogenic and likely pathogenic variants.

It is interesting to note that the frameshift variant accounted for the greatest proportion of pathogenic or likely pathogenic variants (29.4%), although missense mutations were the most common type (64%), followed by frameshifts (14.6%) and nonsense (12.5%) mutations according to the FH mutation database (Figure 12) [27]. In this study, we described a de novo missense mutation of FH as c.1132G > A in two families. Both families presented with uterine myomas. Additionally, the proband in Case 1 also presented with renal cell carcinoma. To trace back to the past medical history, the patient in Case 1 was noticed to have been diagnosed with thyroid cancer at 31 years old. However, as we know, thyroid carcinoma has a high incidence in the general population and a comparatively low mortality rate. Meanwhile, there was no clinical evidence that FH gene mutation was associated with thyroid malignancy, so this could be an incidental event.

The clinical interpretation of this variant was reported as unknown significance in the gene test reports according to the ACMG/AMP (American College of Medical Genetics and Genomics/the Association for Molecular Pathology) 2015 guidelines. This mutation was predicted to be probably pathogenic by Polyphen and deleterious by Provean (Table 1) for protein function, causing change of splice donor of mRNA by prediction tools and likely leading to loss of protein active conformation. Several missense mutations around our mutation spot (c.1132G) were reported, i.e., (c.1138A> G), (c.1130G > A), and (c.1127A > C). In addition, Mutation c.1127A > C was reported pathogenic/likely pathogenic in a few studies [27,29]. FH-Mutant E378K was once reported deleterious in renal cell line April this year, and in vitro experiments constructing a FH-deficient cell line with this mutation further proved the possible pathogenicity of this mutation [30]. The finding led to reasonable speculation that this E378K variant was likely pathogenic, according to the American College of Medical Genetics and Genomics (ACMG) standards and ClinGen guidelines as PS2, PS3_support, PM2_support. PP3, and PP4 [31,32].

We found the FH missense variant c.1132G > A could activate cell proliferation in uterine leiomyoma cell lines as expected. Our study showed mRNA levels of FH-mutant were comparable to that of FH-WT in whole-cell lysates, while protein expression was remarkably decreased in E378K-expressing cells. We supposed that this decrease might be caused by diminished antibody binding due to the aberrant aggregation of mutant proteins, as reported previously. Targeted sequencing of FH-deficient uterine leiomyomas reveals biallelic inactivating somatic fumarase variants and allows characterization of missense variants. Case 1 presented with type II papillary renal cell carcinoma, and research in vitro was executed in the renal cell line to investigate the pathogenicity of this mutation in the kidney, which could serve as a PP3 evidence [30].

UL in patients with FH deficiency can be initially presumed by routine H&E staining due to its distinctive histological characteristics. A study including 108 cases of leiomyoma with bizarre nuclei demonstrated that 62% (67/108) of them presented with an absence of FH on immunostaining, while only 0.2% (1/50) in common uterine leiomyoma and none (0/42) in leiomyosarcomas had loss of FH expression [33], suggesting that leiomyoma with bizarre nuclei was an important pathological characteristic indicating FH-deficient ULs. In our center, ULs with bizarre nuclei would be picked up and further stained for FH protein to screen for potential FH-deficient ULs and sent for genetic counseling to rule out HLRCC. We further investigated the data from the genetic counseling (gene tests results) in our center from Mar-2020 to Mar-2022. Seven families and twenty-two members (female:male 16:6) were diagnosed with HLRCC and agreed to a Sanger test. The total positive rate of gene tests was 54.5% (9/16, 56.3% for females and 3/6, 50% for males). Six of these seven families (85.7%) presented with family associated and eight of nine (8/9, 88.89%) female carriers complained of ULs in their lifetime. The only one carrier who denied history of UL is the mother of the proband in Family 1, and she was 67 years old when the article was submitted. The median age onset of UL is 30 years old (yo) in these eight female carriers (range: 26–40 yo). Siegler et al. [15] collected 22 FH-deficient UL cases diagnosed by immunohistochemistry of FH, and patients’ ages ranged from 25 to 70 years (median 36). In their study, 18 (81.8%) patients had multiple leiomyomas (ranging from 2 to 14 cm) and 0.4% of uterine leiomyomas were proved to be FH-deficient, while none presented as leiomyosarcoma. However, none of these 22 cases had a family history of HLRCC, which is probably the reason why their age onset is older than HLRCC patients in our group (36 yo vs. 30 yo).

Although renal cancer as an early manifestation of HLRCC was less frequent as reported, an appropriate surveillance strategy is enormously necessary for carriers of FH mutations. Fred H. Menko et al. suggested that genetic tests should be considered from the age of 8 to 10 yo. Mutation carriers should be offered an annual MRI with 1–3 mm slices through the kidney to detect micro-lesions [34]. Most of the time, uterine myomas are benign, however, a Finnish study showed that there appeared to be a higher risk of uterine leiomyosarcoma (ULMS) for female HLRCC patients [35]. Early myomectomy and hysterectomy help reduce the incidence of ULMS in HLRCC patients. Currently, there is no evidence of prophylactic hysterectomy following an initial diagnosis of HLRCC. Previous studies have shown that the penetrance of the same gene mutation may be different due to various environmental factors, such as race, BMI, smoking, alcohol, diabetes, etc. [36]. More investigations are needed to study the penetrance and clinical manifestations of different families with the same genetic mutation. There are some limitations to our study, as we failed to achieve family members in Case 2 for Sanger Sequencing to further consolidate the evidence, and long-term follow-up for these two patients was urgently needed to reach a better understanding of HLRCC.

## Figures and Tables

**Table 1:** Prediction of protein function. ^a^ A larger score (close to 1) of PolyPhen2 revealed a probably damaging effect. ^b^ A score smaller than −2.5 indicated a deleterious effect. AA, amino acid.

				PolyPhen2	Provean
Mutation gene		Variant	AA change	Score ^a^	Score ^b^

**Table 2:** The effects on the three-dimensional structure predicted by the tool SWISS-MODEL.

	Pre-Mutation		Post-Mutation	
	Hydrogen Bond	Distance	Hydrogen Bond	Distance
Main Chain	T381 and E378	3.4	T381 and K378	3.4
	M382 and E378	2.9	M382 and K378	3.0
	P374 and E378	3.3	P374 and K378	3.3
	T375 and E378	3.3	T375 and K378	3.2
Side Chain	G144 and E378	2.7	H235 and K378	2.9

## Data Availability

Not applicable.

## Supplementary Materials

The following supporting information can be downloaded at: https://www.mdpi.com/article/10.3390/genes14030744/s1, Figure S1: Cell proliferation in Uterine leiomyoma cell line as control, cell line with FH-WT and FH-MUT (E378K) plasmid transfected; Figure S2: Cell proliferation significantly increased once transfected with FH-MUT (E378K). Table S1: The primer sequences for FH amplification
