# Supplementary material for: A Missense Mutation c.1132G > A in Fumarate Hydratase (FH) Leads to Hereditary Leiomyomatosis and Renal Cell Cancer (HLRCC) Syndrome and Insights into Clinical Management in Uterine Leiomyomata
Source: Genes (Basel). 2023 Mar 18;14(3):744. doi: 10.3390/genes14030744 (PMC10048203; doi:10.3390/genes14030744)
Supplement: Supplementary file 1 [file genes-14-00744-s001.zip › Table S1.pdf]

Gene	Primer Name	Primer Sequence
FH	*FH –QPCR-F*	ATCCACGCTGTTTTGACCTC
	FH-QPCR-R	AGGAATTTTGGCTTGCCATT

Table S1. The primer sequences for FH amplification
