# Supplementary figures and images for: A Missense Mutation c.1132G > A in Fumarate Hydratase (FH) Leads to Hereditary Leiomyomatosis and Renal Cell Cancer (HLRCC) Syndrome and Insights into Clinical Management in Uterine Leiomyomata
Source: Genes (Basel). 2023 Mar 18;14(3):744. doi: 10.3390/genes14030744 (PMC10048203; doi:10.3390/genes14030744)

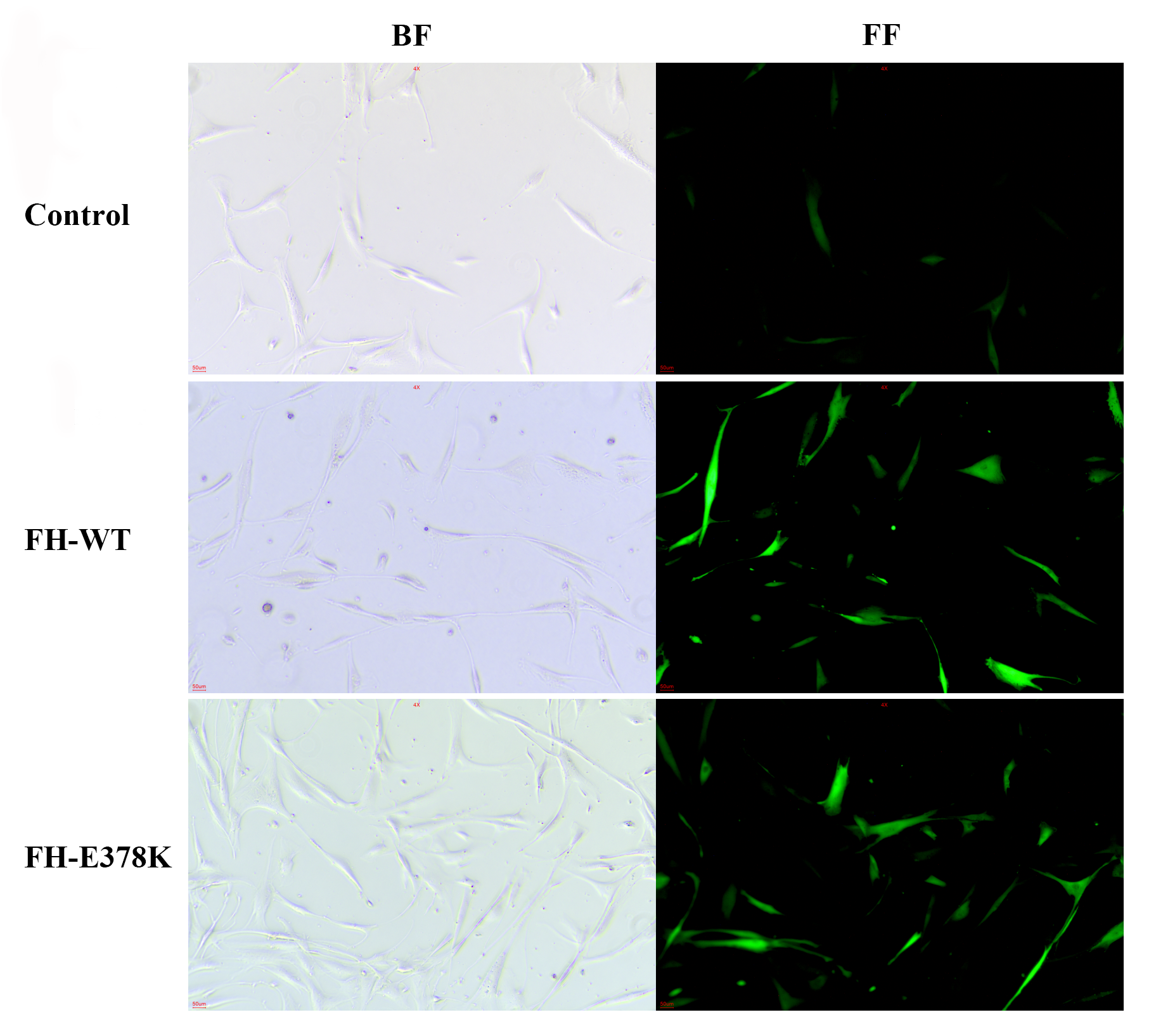

Supplement: Supplementary file 1 [file genes-14-00744-s001.zip › supplementary figS1 .tif]

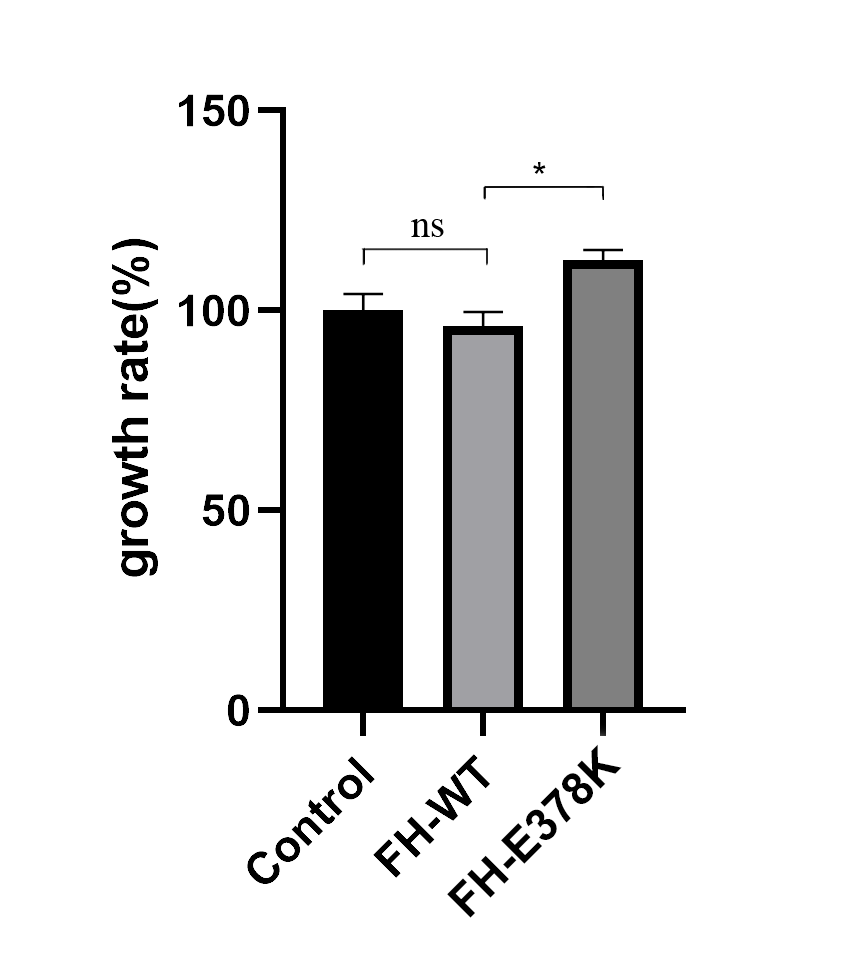

Supplement: Supplementary file 1 [file genes-14-00744-s001.zip › supplementary figS2.tif]
